# Pituitary Spindle Cell Oncocytoma: More than a Grade 1 Tumor?

**DOI:** 10.3390/neurolint17020016

**Published:** 2025-01-22

**Authors:** Jonathan Hammond, Zacharie Gagne, Bojana Mitrovic, Stefano M. Priola

**Affiliations:** 1 Northern Ontario School of Medicine University, Sudbury, ON P3E 2C6, Canada; zgagne@nosm.ca (Z.G.); spriola@nosm.ca (S.M.P.); 2 Department of Pathology, Health Sciences North, Sudbury, ON P3E 2C6, Canada; bmitrovic@hsnsudbury.ca; 3 Department of Neurosurgery, Health Sciences North, Sudbury, ON P3E 2C6, Canada

**Keywords:** spindle cell oncocytoma, pituitary tumors, endoscopic endonasal approach, neuro-oncology

## Abstract

Background/Objectives: Spindle cell oncocytomas (SCOs) of the pituitary gland are rare tumors often misdiagnosed for nonfunctioning pituitary macroadenomas. Although classified as grade 1, they are often challenging in terms of diagnosis and treatment. Pituitary SCOs harbor peculiar features such as hypervascularity and stronger adherence to surrounding structures, with increased risk of hemorrhage, partial resection, and significantly higher recurrence rate. Almost 100 cases have been reported so far. The role of surgery is still crucial for the decompression of the optic chiasm as well as for achieving diagnosis. However, given the higher tendency of recurrence, the role of postoperative radiotherapy has been investigated over the last few years. Case presentation: Here, we reported a case of a 48-year-old female with a pituitary SCO treated at our institution, in which we focused on diagnosis, treatment, and follow-up. Conclusions: This type of tumor presents a challenge related to its higher vascularity and strong adherence to the surrounding structures. Adjuvant radiotherapy is something that should be considered, especially when gross total resection is not achieved, and finally, SCOs require diligent follow-up to monitor for any signs of disease recurrence or progression.

## 1. Introduction

Spindle cell oncocytomas (SCOs) are rare non-neuroendocrine tumors arising from the pituicytes of the posterior pituitary gland. They were included in the World Health Organization (WHO) classification of central nervous system tumors in 2007 after being first described by Roncaroli et al. in 2002 [1,2].

SCOs were previously thought to stem from the adenohypophysis; however, more recent research identified that SCOs are positive for thyroid transcription factor 1 (TTF1) which is only found in pituicytes and not in the folliculostellate cells of the adenohypophysis [3]. In our search from 2002 to 2024, there were only 98 cases published in the literature that highlight the rarity of this type of tumor.

SCOs are often misdiagnosed as pituitary adenomas; nevertheless, they are important to distinguish as they usually have higher vascularity as well as an increased tendency to invade the surrounding structures [4]. This results in a higher recurrence rate, thus requiring more frequent follow-ups. If gross total resection (GTR) is not achieved, 50% of tumors show significant progression requiring extra treatment by two years, recurrence occurs in 20% of people, and even distant metastasis has been reported [4].

Despite these factors, the WHO considers pituitary SCOs a grade 1 tumor with benign behavior [5]. Moreover, although this tumor is considered grade 1, it is important to understand these critical features as it guides treatment and follow-up and will improve patient outcomes.

Due to the low incidence of this neoplasm, the definitive knowledge of the unique clinical signs and symptoms, diagnostic imaging (DI), immunohistochemistry (IHC), and treatment is lacking. In this report and literature review, we aimed to provide further knowledge on these factors to improve the identification and treatment of individuals with SCO. In addition, the role of adjuvant radiotherapy is not fully understood in the treatment of pituitary SCO. However, recent literature demonstrated radiotherapy to be safe and effective for residual SCO after surgery [6]. Radiotherapy will be further assessed in our review of the literature to also help establish a comprehensive understanding of its potential role in the treatment of SCO.

## 2. Case Presentation

We present the case of a 48-year-old female with a two-month history of persistent headaches, mental and physical fatigue, and light sensitivity. Her past medical history includes chronic migraines, fibromyalgia, psoriasis, anxiety, obesity, and obstructive sleep apnea. When looking back, the patient realized that her headache symptoms had changed from her normal migraine symptoms to more frequent albeit less severe frontal pain and aching in the previous months.

**Figure 1:**
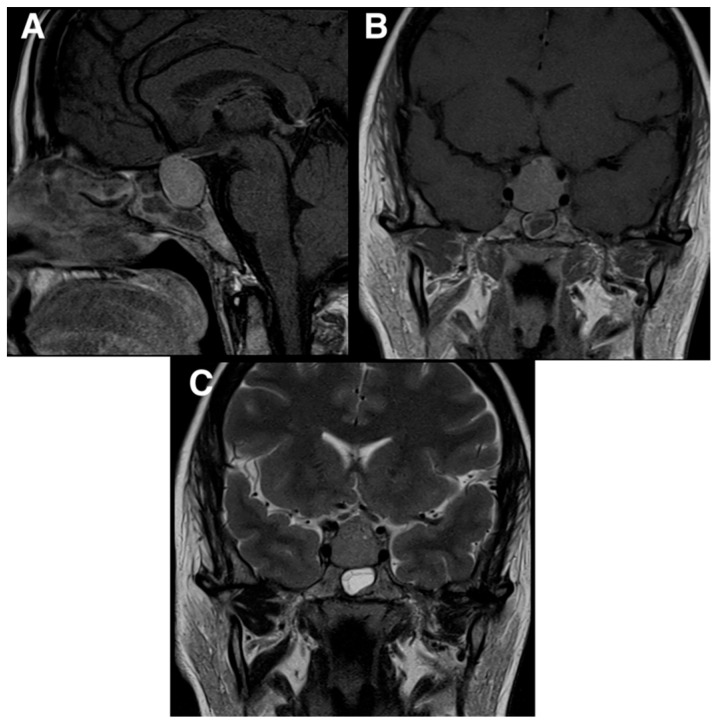
Preoperative MRI scans demonstrating a homogenous mass in the pituitary gland. The MRI also showed that the mass was compressing the optic chiasm and was encasing the carotid arteries. (**A**) T1 MRI sagittal view; (**B**) T1 MRI coronal view; (**C**) T2 MRI coronal view.

Given the persistence of the above-mentioned symptoms, she was investigated with a head computed tomography (CT) scan, which showed a pituitary lesion with sellar and suprasellar extension. For further investigation, she underwent a brain magnetic resonance imaging (MRI) scan that demonstrated a homogenous mass in the pituitary gland (Figure 1). The MRI also showed that the mass was compressing the optic chiasm and was encasing both internal carotid arteries. These findings were thought to be compatible with pituitary macroadenoma.

On physical examination, the patient had no cranial nerve deficits, and her pupils were equal, round, and reactive to light and accommodation. She did not have any focal motor or sensory deficits. She also underwent an ophthalmological assessment that ruled-out papilledema and confirmed normal bilateral visual fields. Lastly, a thorough endocrinological assessment was completed, including a full hormonal panel that demonstrated only mild hyperprolactinemia.

Given the clinical and radiological findings, an elective surgical treatment was recommended and carried out using an endoscopic endonasal transsphenoidal approach. Intraoperatively, the tumor had the usual soft consistency, but it presented stronger adherence with what was thought to be the normal pituitary gland and, therefore, required extra work to separate it. Although no major bleeding was identified, an unusual constant tumor oozing made the procedure more challenging. An apparent GTR of the tumor was achieved and confirmed by the direct visualization of the suprasellar cistern coming down into the sella turcica. For this reason, no adjuvant radiotherapy was performed.

**Figure 2:**
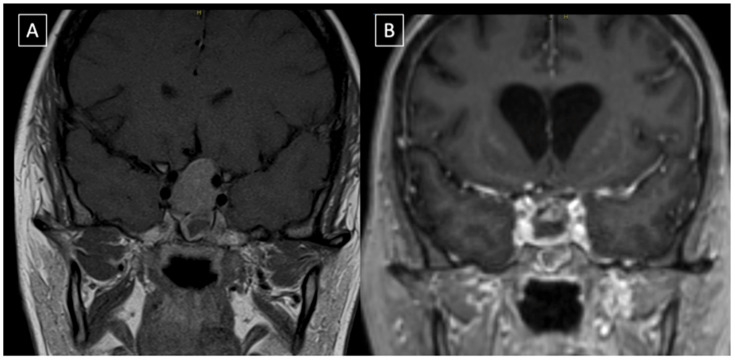
Coronal reconstruction of the pre-op (**A**) and post-op (**B**) post gadolinium T1-weighted images. The post-operative MRI confirms that optic chiasm has been decompressed and gross total resection obtained. There is evidence of minimally enhancing intrasellar material that is likely gel foam used for skull base reconstruction.

The patient had an uneventful postoperative period. She spent two days in the intensive care unit and was then transferred to the floor, where low levels of cortisol and mild diabetes insipidus were diagnosed and treated medically. Post-operative MRI ruled out intraoperative complications and confirmed the decompression of the optic chiasm (Figure 2). She was discharged home on postoperative day seven, neurologically intact.

She was reassessed in the follow-up four weeks after surgery, and no obvious focal deficits were identified. Her headache and light sensitivity had notably improved. A repeat MRI was completed three months postoperatively, and it confirmed the GTR of the SCO; the patient scheduled their next follow-up with repeat MRI at the sixth month mark.

**Figure 3:**
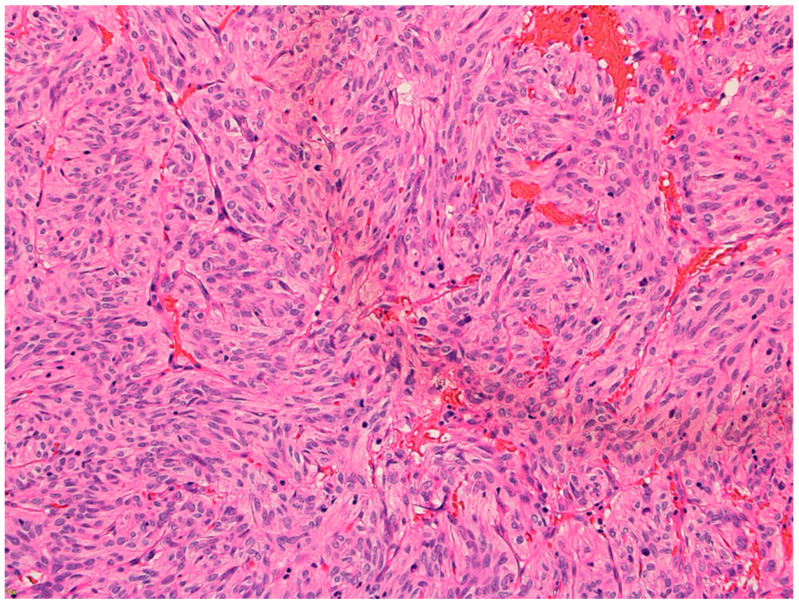
Hematoxylin and eosin (H&E) 20× showing spindled cells with eosinophilic cytoplasm arranged in nests and short fascicles.

**Figure 4:**
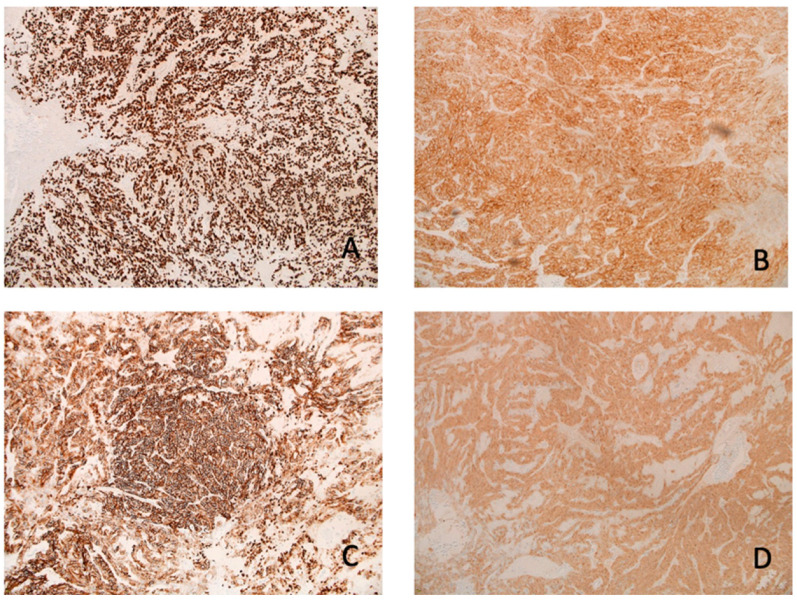
IHC: The neoplastic cells are positive for (**A**) TTF1 (10×), (**B**) synaptophysin (10×), (**C**) CD56 (10×), and (**D**) S100 (10×).

The microscopy examination of the specimen demonstrated fascicles of spindle-cell tumor cells with eosinophilic cytoplasm and elongated nuclei showing moderate pleomorphism (Figure 3). Other areas of tumor cells showed more eosinophilic cytoplasm with clear borders and contained round to oval nuclei with mild pleomorphism. Granular cytoplasm was not seen; however, mitotic figures were present. No tumor necrosis was noted. The tumor cells were diffusely positive for TTF-1, S100, CD56, and synaptophysin (Figure 4). They were also focally positive for epithelial membrane antigen (EMA), glial fibrillary acidic protein (GFAP), CD68, and beta crystallin. There was no immunostaining for pituitary-specific transcription factor-1 (PIT-1) or steroidogenic factor-1 (SF-1).

## 3. Discussion

SCOs are very rare tumors of the posterior pituitary gland that originate from the pituicytes located in the neurohypophysis. They are called SCOs because their cells are spindle shaped under microscopic examination and contain many mitochondria in their cytoplasm [7]. Oncocytomas can occur in many different areas of the body, including kidney, breast, prostate gland, thyroid, and salivary glands [7]. However, they are often only called oncocytomas in these locations as they lack spindle-shaped cells, which are characteristic of the pituitary SCO [7,8].

Unfortunately, pituitary SCOs appear very similar to non-functioning pituitary adenomas in clinical presentation and diagnostic imaging. Yet, they require unique care because of two specific features: increased vascularity and increased fibrotic adherence to surrounding structures.

Additionally, SCOs’ increased vascularity has the potential to cause significant bleeding during surgery. Borges et al. described a case of recurring subclinical tumor bleeding that happened in a recurrent SCO [9,10]. In the same manuscript, Borges reported that nearly one-third of assessed cases showed significant intraoperative bleeding, and almost half of these cases demonstrated that the tumor was highly vascular [9,10]. Cases of spontaneous tumor hemorrhage were also described.

Careful tumor dissection and the strategic use of cottonoids with hemostatic agents is crucial when dealing with any tumor of the posterior pituitary gland, especially SCO. Also, SCOs have an elevated risk of progression or recurrence because of the adherent characteristics of the tumor. In a study by Hasegawa et al., it was found that GTR was only achieved in 24% of cases mainly because of those tumor features [5]. These characteristics make the recurrence of SCOs very likely, with 50% of tumors showing significant progression if GTR is not attained and radiotherapy is not added [5].

There are also case reports of SCOs managed transcranially. In fact, in cases with extensive suprasellar extension, this approach can allow for more intraoperative maneuverability to deal with significant bleeding and provide better accessibility to all areas of the tumor [10].

Oftentimes, partial resection is all that can be accomplished, leading to the growth of any residual tumor. In cases like this and with general tumor recurrence, interdisciplinary support should be pursued and thought given to both reoperation as well as adjuvant radiotherapy [10].

The role of preoperative radiotherapy is still being debated; Hasegawa et al. found in their meta-analysis that preoperative radiotherapy did not have an impact on those who achieved GTR [5]. There was also no statistical difference between the non-GTR group that received preoperative radiotherapy and the non-GTR group that did not receive preoperative radiotherapy. However, in patients who do not achieve GTR, postoperative radiotherapy should be considered as it has shown promising results in controlling tumor progression [11].

In our review of the literature (Table 1), we performed a search from 2002 to 2024 using databases such as PubMed, Google Scholar, and ScienceDirect to identify all published articles reporting pituitary SCO, which totaled 98 cases. Primary search terms included pituitary spindle cell oncocytoma, pituitary tumors, and posterior pituitary tumors. Case reports, case series, and original articles were included, whereas articles not presenting new cases or unpublished material were excluded. Article references were also hand searched to ensure no reports were missed. Each case was then analyzed for specific data points, including age, sex, clinical presentation, diagnostic imaging, IHC, surgical approach, use of adjuvant radiotherapy, and general outcomes. The data were then summarized into a structured table which was used to draw conclusions on significant topics like recurrence rates and the effectiveness of radiotherapy, as well as to present all cases reported in the literature thus far. All articles were screened for relevance by the authors, and any discrepancies were resolved through collaborative discussion.

The mean age of all the patients with pituitary SCO was found to be fifty-seven years. Regarding any difference between sex, forty-seven patients were female, and fifty-one patients were male. Of particular importance, we found that there was recurrence or tumor growth in 31% of patients with pituitary SCO who did not receive adjuvant radiotherapy. Meanwhile, only 18% of patients who did receive adjuvant radiotherapy experienced tumor recurrence or progression. This information on adjuvant radiotherapy shows that it has the potential to be very useful in achieving tumor stability and decreases the chances of progression and need for further operations. Akyoldas et al. reported five cases that utilized radiotherapy, all of which showed the tumors to be stable at follow-up. Also, in this study, gamma knife radiosurgery was used each time and reported a median tumor margin dose of 12Gy and a median maximal dose of 24Gy [6].

These rates of recurrence/progression underscore the importance of continued surveillance with these tumors. Many of the reports noted how partial resection was all that could be achieved due to the highly vascular nature of the tumor. However, recurrence was even found in cases that appeared to achieve GTR.

Our case specifically highlights the importance of being aware of the high vascularity of these tumors due to their increased risk of bleeding during surgical resection. If a SCO is suspected/identified, measures can be put into place to prepare for increased bleeding, such as careful tumor dissection and strategic use of cottonoids with hemostatic agents in addition to blood products typed and matched if they are needed. Special consideration is also needed regarding the follow-up plan for patients with SCO due to their increased rate of recurrence.

## 4. Conclusions

SCOs are rare tumors of the posterior pituitary gland that have many unique features that require specific treatment and follow-up. Although this tumor presents similarly to a pituitary adenoma, there are marked differences in the physical appearance of the tumor, IHC, and follow-up required. From a surgical perspective, the challenge is related to higher vascularity and stronger adherence to the surrounding structures. This makes the surgery itself more difficult and a GTR less likely, with a higher recurrence rate.

To date, the use of radiotherapy was not well established. However, our review does provide encouraging results that post-operative radiotherapy has the potential to minimize tumor progression and increase tumor stability. Only 18% of the patients who had a pituitary SCO and received adjuvant radiotherapy developed recurrence/progression compared to 31% of patients who did not receive adjuvant radiotherapy.

In our opinion, radiotherapy should definitely be considered, especially when GTR is not achieved. In our case, a GTR was achieved; thus, we decided to proceed without radiotherapy and with closer follow-up appointments to identify any early sign of recurrence. Closer follow-up appointments are also very necessary as many patients develop recurrence or tumor progression, sometimes despite GTR. These high rates of tumor recurrence/progression underscore the need for careful and frequent monitoring.

## Figures and Tables

**Table 1:** Review of the literature (2002–2024).

	Age	Sex	Clinical Presentation	Diagnostic Imaging	IHC	Surgery	Post-Op Radiotherapy	Outcomes
Hsieh et al., 2024 [8]	69	Male	Facial pain and mild decrease in visual acuity	Sellar and suprasellar lesion	S-100 protein, EMA and TTF-1	Transsphenoidal partial resection Tan-pink, soft, and well-circumscribed mass	Not described (ND)	Uneventful post-op period, follow-up ND
	68	Female	Bi-temporal visual defect	Large sellar mass with mass effect over brainstem and optic chiasm	S-100 protein, EMA(E29) and TTF-1	Transsphenoidal partial resection	Yes 500 ×5 cGy	2-year follow-up MRI showed partial size decreased of the tumor
Joshi et al., 2024 [2]	71	Male	ND	Sellar mass, 4 mm	ND	Transsphenoidal resection	ND	8-week follow-up no headache or visual disturbances
Chang et al., 2023 [11]	31	Male	Bilateral vision loss—temporal defect	Sellar mass with suprasellar extension	EMA, S-100, and TTF-1	Transsphenoidal total resection, mass was yellow and soft with easy bleeding	ND	ND
Kunihiro et al., 2023 [4]	53	Male	Headache and diplopia	Sellar mass with suprasellar extension	S-100, TTF-1 and vimentin	Transsphenoidal total resection, mass was yellow and soft significant bleeding	No	No enlargement of residual tumor at 1-year follow-up
Shimizu et al., 2022 [12]	40s	Female	Headache	Lesion with cystic area in the intra-suprasellar region	TTF-1, S-100 protein, vimentin, GFAP, EMA	Transsphenoidal resection, tumor was fibrous and easy to bleed	ND	No recurrence at 3 years
Tena-Suck et al., 2022 [13]	66	Male	Headache, chiasmatic syndrome, and bitemporal hemianopsia	Sellar lesion and a left frontal cystic lesion	Imentin, pit-1, PTTG-1, TTF-1, S100	ND	ND	ND
Abdulrazeq et al., 2021 [14]	74	Female	Persistent headaches and vertigo	Sellar mass with lateral extension	Vimentin, annexin A1, S-100, and TTF-1	Transsphenoidal partial resection	No	No progression at 6-month follow-up
Hasegawa et al., 2021 [5]	49	Female	Galactorrhea, numbness, headache	Sellar mass with slight suprasellar extension	S-100, TTF-1, and GFAP	Transsphenoidal partial resection Grayish, firm, markedly adhesive	No	Radiosurgery at 20 months for slight tumor progression, MRI at 39 months showed tumor stability
	55	Male	Fatigue, muscle weakness, weight loss	Sellar mass with suprasellar extension	S-100 protein, TTF-1	Transsphenoidal partial resection Firm, hypervascular	No	GK at 12 months for residual
	78	Male	Fatigue, visual deficit, hyponatremia	Sellar mass with suprasellar extension	S-100 protein, TTF-1	Transsphenoidal partial resection	No	GK at 7 months for residual
	59	Female	Fatigue, hyponatremia	Sellar mass with suprasellar extension	S-100 protein, TTF-1	Transsphenoidal partial resection	No	GK at 7 months for residual
	56	Male	hyponatremia	Sellar mass with suprasellar extension	S-100 protein, TTF-1	Transsphenoidal partial resection	No	Transsphenoidal surgery at 77 months for recurrence
	66	Female	Nausea, vomiting, weight loss	Sellar mass with suprasellar extension	S-100 protein, TTF-1	Transsphenoidal gross total resection	No	GK at 31 months for recurrence
Kim et al., 2021 [15]	42	Female	Bitemporal hemianopsia	Sella mass with suprasellar extension	Vimentin, EMA, S-100 protein, TTF-1, and galectin-3	Transsphenoidal partial resection Highly vascular, pale-yellow and solid	No	Regrown mass at 4 months requiring a second surgery
Kottangal et al., 2021 [16]	61	Female	Hyponatremia and temporal field cut	Sellar mass with suprasellar extension	EMA, S-100 protein, TTF-1	Transsphenoidal resection Grey-white, friable, and soft	ND	ND
Taka et al., 2021 [17]	75	Male	Bitemporal hemianopsia	Sellar mass with suprasellar extension	ND	Transsphenoidal gross total resection	ND	Follow-up on the eighth postoperative day showed improvement of peripheral vision.
Tariciotti et al., 2020 [18]	64	Female	Bitemporal hemianopia, hyposmia, headache	Sellar mass with suprasellar extension causing hydrocephalus	S100, neuron-specific Enolase, TTF-1	Transsphenoidal partial resection heavy intraoperative bleeding	ND	Recurrence at 5 months, partial resection surgery again with close follow-up
Samadian et al., 2020 [19]	8	Male	VD	Sellar and suprasellar mass	EMA, vimentin, and S-100	Transsphenoidal gross total resection	No	No signs of progression at 2 year follow-up
Borg et al., 2020 [20]	55	Female	Dizziness	Sellar mass with suprasellar extension	TTF-1, EMA and S-100	Subtotal transsphenoidal resection	No	Progression at 8 years requiring radiotherapy
	71	Male	Incidental	Sellar mass	TTF-1, EMA, S100	Subtotal transsphenoidal resection Tough, grey and gelatinous.	ND	Residual remained stable in size for four years
Li et al., 2020 [21]	57	Male	Visual defect and neck pain	Sellar mass	EMA, TTF-1, S100, vimentin, annexin1, and SSTR2	Total transsphenoidal resection	ND	Patient recovered well and had return of normal vision
Chainey et al., 2020 [22]	49	Male	Confusion, memory loss, and increased drowsiness	Sellar mass with suprasellar extension	ND	ND	ND	Recurrence/growth at 4 and 5 years requiring two further resections
Akyoldas et al., 2019 [6]	55	Female	VD	Sellar mass	ND	Transsphenoidal resection	Yes 14 Gy	Stable at 84 months
	41	Male	Loss of body hair, infertility	Sellar mass	ND	Transsphenoidal resection	Yes 12 Gy	Stable at 51 months
	61	Male	Headache—VD	Sellar mass	ND	Transsphenoidal resection	Yes 14 Gy	Stable at 47 months
	50	Male	VD	Sellar mass	ND	Transsphenoidal resection	Yes 14 Gy	Stable at 41 months
	56	Male	VD	Sellar mass	ND	Transsphenoidal resection	Yes 14 Gy	Stable at 36 months
Sollfrank et al., 2019 [23]	38	Female	ND	Mass in right parasellar region	ND	History of six surgical excisions, radiation and chemo radiation for local recurrence of SCO. Most recent treatment was vemurafenib (BRAF inhibitor)	No	Stable—no progression on BRAF inhibitor at two-year follow-up
Yip et al., 2019 [24]	28	Female	Severe headache and blurred vision, decreased right visual acuity, visual field defects, low cortisol	Sellar mass with suprasellar extension	TTF-1, EMA, Annexin A1	Transsphenoidal total resection mass was yellow and soft	ND	ND
Cole et al., 2019 [25]	64	Male	Headache, fatigue, vision changes, Endocrine abnormality	Sellar mass	TTF-1, EMA, GFAP, S100	Transsphenoidal total resection	ND	No recurrence at follow-up
	70	Male	Headache, fatigue, vision changes, Endocrine abnormality	Sellar mass	TTF-1, EMA, GFAP, S100	Transsphenoidal total resection	ND	No recurrence at follow-up
	27	Female	Endocrine abnormality	Sellar mass	TTF-1, EMA, GFAP, S100	Transsphenoidal total resection	ND	No recurrence at follow-up
Guerrero-Pérez et al., 2019 [3]	74	Female	VD	Sellar mass	TTF-1, S100, VIM	None	ND	ND
	69	Female	Weakness	Sellar/suprasellar mass	TTF-1, S100, VIM, CD56	Total transsphenoidal resection	ND	ND
	74	Female	Nausea, vomiting and confusion	Sellar/suprasellar mass	TTF-1, S100, VIM, GFAP	Subtotal transsphenoidal resection	ND	ND
	60	Male	VD	Sellar/suprasellar mass	ND	Subtotal transcranial resection	ND	ND
	60	Male	VD	Sellar/suprasellar mass	ND	Subtotal transsphenoidal resection	ND	ND
	62	Female	VD	Sellar mass	ND	Total transsphenoidal resection	ND	ND
Witte et al., 2018 [26]	61	Male	Headaches, bilateral retrobulbar pressure sensation, light sensitivity, and drowsiness	Sellar mass	VIM, Gal3, Bcl-2	Transsphenoidal partial resection	No	Three reoperations were required for multiple tumor reccurences along with radiation and chemotherapy
Larsen et al., 2018 [10]	66	Female	Dizziness, nausea, diaphoresis	Sellar mass	EMA, TTF-1, S100	Transsphenoidal partial resection	ND	Stable residual tumor at 100 months
	50	Male	Dizziness	Sellar mass	EMA, TTF-1, S100	Transsphenoidal partial resection	ND	Stable residual tumor at 30 months
	63	Male	VD	Sellar mass	TTF-1, S100	Transsphenoidal partial resection	ND	Repeat resection (3 months after surgery); craniotomy for further progression (42 months post repeat procedure)
	59	Female	VD	Sellar mass	EMA, TTF-1, S100	Transsphenoidal partial resection	ND	Recurrence at 6 years, treated w/ repeat resection, GK; stable at 79 months
	77	Male	VD	Sellar mass	EMA, TTF-1, S100	Transsphenoidal gross total resection	ND	Stable after GTR (12 mos)—no complications
	56	Female	Eyeball heaviness, nausea, dizziness	Sellar mass	EMA, TTF-1, S100	Transsphenoidal partial resection	ND	Radiotherapy for residual tumor; stable at 38 months
Gupta et al., 2018 [27]	28	Female	Bilat vision loss, headaches, amenorrhea, galactorrhea	Sellar mass with suprasellar extension	EMA, S-100, and TTF-1	Transsphenoidal resection	ND	At 7 month follow-up, she was free of headache and galactorrhea and had a normal vision
Yoshida et al., 2018 [28]	69	Female	Bitemporal hemianopsia	Sellar tumor with suprasellar extension	TTF-1	Subtotal transsphenoidal resection. Tumor was extremely hypervascular	ND	No regrowth found at 6 months
Nagata et al., 2018 [29]	40	Female	VD	Sellar and suprasellar mass.	EMA, S-100, TTF-1, GFAB, Vimentin	Total transsphenoidal resection Hypervascularized lesion	No	ND
Sosa et al., 2018 [30]	60	Male	VD, fatigue, decreased libido, and erectile dysfunction for the past 8 months	Sellar mass	Vimentin, S100 protein, and TTF-1	Transsphenoidal partial resection	No	Radiotherapy after 5-month follow-up No residual or recurrent tumor was observed at 4-year follow-up
Xie et al., 2017 [31]	60	Male	Nausea, vomiting, fatigue and syncopal episodes	Sellar and suprasellar mass	Vimentin, S-100, EMA and TTF-1	Transsphenoidal surgical resection Vascular whitish-yellow mass with soft consistency	No	No evidence of tumor recurrence after 18-month follow-up
Rafiq et al., 2017 [32]	61	Male	VD, fatigue and weight loss	Sellar lesion with compression of the optic chiasm	Vimentin, S-100, EMA and TTF-1	Subtotal trans sphenoidal resection Firm, fibrous and greyish	No	3-year follow-up, scan showed tumor progression requiring surgery, GTR was achieved
	69	Female	Rapidly progressive visual deterioration	Large sellar tumor with a suprasellar extension	S-100, EMA and TTF-1	Transphenoidal resection Firm, greyish in color, moderately vascular	No	After 6-month follow-up, no progression
Osman et al., 2017 [33]	56	Male	Headache, vomiting, neck pain, back pain, and reduced level of consciousness	Sellar and suprasellar mass	Vimentin, S-100, EMA and TTF-1, GFAP	Sub-frontal craniotomy. Profuse bleeding limited the surgical resection.	Yes	No tumor recurrence at six-month follow-up
Manoranjan et al., 2017 [34]	60	Male	Temporal loss in both visual field quadrants of his left eye	Sellar and suprasellar mass	S100, Vimentin, Bcl2, CD56, TTF-1	Subtotal transnasal transsphenoidal resection	ND	No tumor progression at most recent follow-up
Sali et al., 2017 [35]	64	Male	Drooping of the left eyelid for 2 months and left temporal hemianopia	Sellar and suprasellar lesion	S100, synaptophysin, EMA, TTF-1	Transsphenoidal resection	ND	ND
Billeci et al., 2017 [36]	61	Male	Headache and clinical signs of mild hypopituitarism	Sellar-suprasellar mass involving the sphenoidal sinus and chiasmatic cistern.	Vimentin, S-100, TTF-1	Subtotal transnasal transsphenoidal resection. Tumor was firm and highly vascularized	ND	After 14 months from the second surgery, no increase in residual tumor size has been documented
	65	Female	VD and severe headache	sellar-supraasellar mass with a size of	Vimentin, S-100, TTF-1	Subtotal transnasal transsphenoidal resection. The tumor was firm, fibrotic and highly vascularized	ND	No documented recurrences after 28 months of follow-up
Kong et al., 2017 [37]	30	Male	Headaches, fatigue, diplopia, and impaired visual field and acuity for 6 months	Suprasellar and parasellar leson	Vimentin, CD68, CD34, Nestin, GFAP, Desmin, SMA, AE1/AE3, and S-100 protein	Subtotal transnasal transsphenoidal resection. Hypervascular	ND	Two more resections were done for tumor recurrence/progression
Hagel et al., 2017 [38]	65	Female	ND	ND	S100, CD68, TTF, Vimentin, neuron specific enolase	ND	ND	ND
	41	Female	ND	ND	S100, CD68, TTF, GFAP	ND	ND	ND
	64	Female	ND	ND	Vimentin, EMA, S100, TTF	ND	ND	ND
	53	Male	ND	ND	Vimentin, EMA, MAP2, S100, CD68, TTF	ND	ND	ND
Custodio et al., 2016 [39]	60	Male	NVD/Fatigue, hyponatremia, panhypopituitarism, low cortisol	Sellar mass with suprasellar extension	Vimentin, EMA, S-100, and TTF-1	Transsphenoidal partial resection yellow white mass significant bleeding	ND	No growth at 18 months
Hasiloglu et al., 2016 [40]	40	Male	Panhypopituitarism	Intra-suprasellar mass and enlargement of the sella turcica	Vimentin, galectin-3, EMA and S-100	Transsphenoidal partial resection	No	Recurrence after one year, repeat surgery
	60	Male	Headache, visual blurring	Intra-suprasellar mass and enlargement of the sella turcica	Vimentin, galectin-3, EMA and S-100	Transsphenoidal partial resection	No	ND
	55	Male	Headache, visual blurring	Intra-suprasellar mass and enlargement of the sella turcica	Vimentin, galectin-3, EMA and S-100	Transsphenoidal partial resection	No	ND
Guadagno et al., 2016 [41]	77	Male	Headache and temporal hemianopsia of the right eye	Sellar mass with suprasellar extension	EMA, Vimentin, S100 protein, Galectin-3, and TTF-1, and focal positivity for Bcl-2	Transsphenoidal total resection	ND	14-month follow-up with no evidence of recurrence
Vuong et al., 2016 [42]	70	Male	Visual disturbance and headache	Sellar-suprasellar lesion	Vimentin, TTF-1, EMA and galectin-3	Transsphenoidal partial resection	ND	Tumor recurrence not detected at first follow-up exam
Zygourakis et al., 2015 [43]	55	Female	Headaches	Sellar mass	AMA, EMA, S100, GFAP, TTF1	Transsphenoidal resection	ND	No reccurence on follow-up
	31	Female	Bitemporal hemianopsia	Sellar and suprasellar lesion	TTF1, EMA and AMA	Transsphenoidal partial resection	No	MRI at six months showed stable residual tumor
Mu et al., 2015 [44]	35	Female	Amenorrhea, lactation, decreased visual acuity	Suprasellar round mass	Vimentin, EMA, S-100 and TTF-1	Frontotemporal craniotomy, complete removal	ND	No recurrence at 21 months
	62	Female	No clear symptoms or signs	Suprasellar mass	Vimentin, EMA, S-100 and TTF-1	Right trans-pterional craniotomy, complete removal	ND	No recurrence at 15 months
Rotman et al., 2014 [45]	88	Male	Fatigue and decreased vision	2-cm intrasellar mass with suprasellar extension	Vimentin	Transsphenoidal partial resection	ND	ND
Fujisawa et al.k., 2012 [46]	68	Male	Unsteady gait, malaise, depression, panhypopituitarism and visual field defects	Sellar mass with suprasellar extension	EMA, S-100, and vimentin	Transsphenoidal partial resection	Yes 50 Gy	1.5-year follow-up showed tumor progression, partial resection again with close follow-up
Alexandrescu et al., 2012 [47]	24	Female	Headaches, amenorrhea and left superior visual field disturbance of the left eye	Sellar mass	EMA, S100, vimentin	Sublabial trans-septal approach, total resection Yellow and more firm	ND	No recurrence at 6 months
Singh et al., 2012 [48]	68	Male	Head and visual deficits	Sellar–suprasellar mass	Vimentin, S100, and EMA	Sublabial transsphenoidal partial resection	ND	ND
Ogiwara et al., 2011 [49]	39	Male	Headache, loss of stamina and libido, bitemporal hemianopia, and polyuria	Suprasellar lesion with the compression of the optic nerves	TTF-1, EMA, S-100, and galectin-3	Transcranial partial resection	Yes 5040 cGy	Recurrence at 9 months requiring repeat surgery. Transsphenoidal resection for second recurrence. No evidence of recurrence at 1-year follow-up since last surgery.
Romero-Rojas et al., 2011 [50]	42	Female	Oligomenorrhea	Sellar mass	Vimentin, S10, EMA, and antimitochondrial antibody MU213-UC clone 131	Transsphenoidal resection	ND	ND
Vajtai et al., 2011 [51]	55	Female	Panhypopituitarism	Intra- and suprasellar tumor	S100 protein, vimentin, Bcl-2, and TTF-1	Transsphenoidal total resection	ND	ND
Mlika et al., 2011 [52]	45	Female	Headache and visual deficit	Pituitary mass with suprasellar extension	Vimentin, S100, EMA and TTF-1	Transsphenoidal total resection	No	No recurrence at 3 months
Borges et al., 2011 [9]	70	Female	Visual deficit in left eye	Intrasellar and suprasellar mass	Vimentin and S100	Sublabial gross total, transsphenoidal resection	ND	Recurrence requiring second transsphenoidal subtotal resection
Matyja et al., 2010 [53]	63	Female	Headache, vertigo, fatigue, bitemporal hemianopsia, nausea/vomiting and sleepiness	Pituitary mass with suprasellar extension	Vimentin, S100, EMA	Transsphenoidal total resection	ND	No recurrence at 28-month follow-up
	65	Female	Pituitary hormone insufficiency	Sellar mass with suprasellar extension	Vimentin, S100, EMA and galactin-3	Frontal right craniotomy gross section	ND	No recurrence at twenty months
Demssie et al., 2011 [54]	59	Male	Bitemporal hemianopsia, panhypopituitarism, weight loss, vomiting and tiredness	Sellar mass with suprasellar extension	S100 and EMA	Transsphenoidal partial resection	ND	Recurrence at 9 months requiring repeat surgery with radiotherapy
Borota et al., 2009 [55]	55	Female	Weight loss, headaches	Sellar mass	Vimentin, S100 and galactin-3	Transsphenoidal partial resection	No	Growth of the tumor at 1 year requiring radiotherapy
Coiré et al., 2009 [56]	63	Female	Weight loss, anorexia, fatigue, headaches, visual deficits	Large sellar and suprasellar lesion, 3 cm in diameter	S100, vimentin, EMA and gal-3	Transsphenoidal resection	No	Growth at five months requiring second surgery and radiotherapy
Farooq et al., 2008 [57]	76	Male	Weakness and headache	Sellar mass	S100 and EMA	Transsphenoidal partial resection	Yes	No growth at 2-year follow-up
Vajtai et al., 2006 [58]	48	Female	Fatigue, exercise intolerance, and visual deficits	Sellar mass with supra and parasellar extension	S100 protein, vimentin, and EMA	Transsphenoidal total resection	No	No recurrence at 16-year follow-up
Dahiya et al., 2005 [59]	26	Male	Headache, blurred vision in the right eye, nausea, vomiting and impotence	Sellar mass	S100 and EMA	Pterional craniotomy with subtotal resection	Yes 54 Gy over a period of 2 months	No growth over 7 years
	55	Female	Headache and visual deficits	6.5 × 3.3 × 4 cm sellar and parasellar mass	S100 and EMA	Transsphenoidal total resection	ND	No recurrence at 6 months
Kloub et al., 2005 [60]	71	Female	Bilateral vision loss	Sellar mass	Vimentin, S-100 protein, neuron specific enolase, and EMA	Transsphenoidal resection	ND	Recurrence at 3 years
	76	Male	Epistaxis	Sellar mass	EMA and S-100	Transsphenoidal resection	ND	Recurrence at 3 years (repeat surgery and radiotherapy) and 10 years (third resection surgery)
Roncaroli et al., 2002 [1]	Mean age was 62	Female	hypopituitarism	Sellar mass with suprasellar extension	S100, vimentin, EMA and gal-3	Transsphenoidal gross total resection	ND	No recurrence at follow-up (average follow-up of 35.4 months)
	-	Female	hypopituitarism	Sellar mass with suprasellar extension	S100, vimentin, EMA and gal-3	Transsphenoidal gross total resection	ND	No recurrence at follow-up
	-	Male	hypopituitarism	Sellar mass with suprasellar extension	S100, vimentin, EMA and gal-3	Transsphenoidal gross total resection	ND	No recurrence at follow-up
	-	Male	hypopituitarism, visual deficit	Sellar mass with suprasellar extension	S100, vimentin, EMA and gal-3	Transsphenoidal gross total resection	ND	No recurrence at follow-up
	-	Male	hypopituitarism, visual deficit	Sellar mass with suprasellar extension	S100, vimentin, EMA and gal-3	Transsphenoidal gross total resection	ND	No recurrence at follow-up

## Data Availability

The original contributions presented in the study are included in the article, and further inquiries can be directed to the corresponding author.

## Abbreviations

The following abbreviations are used in this manuscript:

IHC	Immunohistochemistry
SCO	Spindle-cell oncocytoma
CT	Computed Tomography
MRI	Magnetic resonance imaging
TTF-1	Thyroid Transcription Factor-1
EMA	Epithelial Membrane Antigen
NVD	Nausea, vomiting, and diarrhea
F/U	Follow-up
VS	Vision
VD	Visual defect
PIT-1	Pituitary-specific positive transcription factor-1
PTTG-1	Pituitary Tumor Transforming Gene-1
GFAP	Glial Fibrillary Acidic Protein
Gal3	Galectin-3
Bcl2	B-cell lymphoma 2
TSR	Transsphenoidal resection
GK	Gamma Knife
Gy	Gray
ND	Not described
AMA	Anti-mitochondrial Ab
